# Evaluation of complex integrated care programmes: the approach in North West London

**DOI:** 10.5334/ijic.974

**Published:** 2013-03-08

**Authors:** Felix Greaves, Yannis Pappas, Martin Bardsley, Matthew Harris, Natasha Curry, Holly Holder, Ian Blunt, Michael Soljak, Laura Gunn, Azeem Majeed, Josip Car

**Affiliations:** Department of Primary Care and Public Health, Reynolds Building, Charing Cross Campus, Imperial College London, London, UK W6 8RP; School of Health Sciences, City University London, UK EC1VOHB; The Nuffield Trust, London, UK W1G 7LP; Department of Primary Care and Public Health, Imperial College London, UK W6 8RP; The Nuffield Trust London, UK W1G 7LP; The Nuffield Trust London, UK W1G 7LP; The Nuffield Trust London, UK W1G 7LP; Department of Primary Care and Public Health, Imperial College London, UK W6 8RP; Department of Primary Care and Public Health, Imperial College London, UK W6 8RP; Department of Primary Care and Public Health, Imperial College London, UK W6 8RP; Department of Primary Care and Public Health, Imperial College London, UK W6 8RP

**Keywords:** integrated, evaluation, delivery, complexity

## Abstract

**Background:**

Several local attempts to introduce integrated care in the English National Health Service have been tried, with limited success. The Northwest London Integrated Care Pilot attempts to improve the quality of care of the elderly and people with diabetes by providing a novel integration process across primary, secondary and social care organisations. It involves predictive risk modelling, care planning, multidisciplinary management of complex cases and an information technology tool to support information sharing. This paper sets out the evaluation approach adopted to measure its effect.

**Study design:**

We present a mixed methods evaluation methodology. It includes a quantitative approach measuring changes in service utilization, costs, clinical outcomes and quality of care using routine primary and secondary data sources. It also contains a qualitative component, involving observations, interviews and focus groups with patients and professionals, to understand participant experiences and to understand the pilot within the national policy context.

**Theory and discussion:**

This study considers the complexity of evaluating a large, multi-organisational intervention in a changing healthcare economy. We locate the evaluation within the theory of evaluation of complex interventions. We present the specific challenges faced by evaluating an intervention of this sort, and the responses made to mitigate against them.

**Conclusions:**

We hope this broad, dynamic and responsive evaluation will allow us to clarify the contribution of the pilot, and provide a potential model for evaluation of other similar interventions. Because of the priority given to the integrated agenda by governments internationally, the need to develop and improve strong evaluation methodologies remains strikingly important.

## Introduction

Integrated care refers to many different models of care [1] yet underlying these is a model where the patient’s journey through the system of care is made as simple as possible. Integration of care is expected to improve quality of care, patient safety and cost effectiveness [2–4]. As a result, the English Department of Health has been actively encouraging integration of care within local health economies, and included a duty to encourage integration in national legislation [5].

Getting integrated care right, and then demonstrating its effectiveness, is a clinical, organisational and research challenge. Several local attempts to introduce integrated care in the English National Health Service (NHS) have been tried, with limited success [6]. Results of the national Integrated Care Pilot programme showed that despite some improvements in process, and staff perceptions that care was being integrated, the pilot programmes achieved only limited improvements in clinical effectiveness and reduction in cost, and had little effect on patient satisfaction [7].

For the integrated care agenda to proceed, robustly evaluated examples in real-world conditions are needed to examine effectiveness, justify investment and consider their potential for implementation on a large-scale. In this paper, we describe a comprehensive evaluation approach that assesses multiple aspects of a large and complex integrated care intervention in London known as the North West London Integrated Care Pilot (NWL ICP). The evaluation involves several work streams that assess the broad aims of the pilot including how it fits within the wider health economy, its impact on clinical outcomes and cost, and the patients’ and professionals’ experience of integrated care.

## The intervention

The aim of the NWL ICP is to improve care for 15,000 people with diabetes and 22,000 people over the age of 75 in northwest London. It seeks to improve the quality of care yet at the same time reduce emergency admissions and the overall cost of care. It is a large, complex intervention covering over one hundred general practices, five local authorities, two mental health trusts, five primary care trusts, two acute hospital trusts and two voluntary organisations. The population covered is typical of inner London, with pockets of extreme affluence and deprivation side by side in an area of high population density.

As described in Harris et al. [8], the approach taken in NWL ICP contains several interventions including: risk stratification using the combined predictive model; care planning across care settings; multi-disciplinary group (MDG) meetings; new financial incentives for participating organisations; and a new information technology (IT) system to facilitate sharing of information and patient records between providers (see Box 1). The MDG meetings are designed to deliver joined-up care by bringing different health care professionals together (including GPs, hospital specialists, mental health care, community nursing, social care, and other allied health care professionals) to discuss the management of those with diabetes or older than 75 years that have been identified as having the most complex needs. Care plans are agreed in these meetings, which can then be monitored using the IT tool. The MDGs also have a secondary aim to improve interaction between primary, community, social and hospital care teams, hopefully leading to enhanced delivery models. The IT tool has been developed for the intervention and allows the various partner organizations to share, store, and analyse patient data. In particular, it allows referral support, performance management and risk management to take place, combining data from the various organizations in a central secure database. The NWL ICP is governed by an unincorporated association of its constituent organisations, with a regular management board chaired by an independent representative. It has a small dedicated management team to run the pilot on a day-to-day basis.

## The logic of the evaluation

This paper describes the evaluation methodology for the NWL ICP, a significant integrated care program within a purchaser-provider split, taxpayer-funded national health system. Central in developing the evaluation plan was to recognise the complexity of the intervention in clinical, financial, strategic and political contexts. In particular, we see that the intervention targets “several integrating components” of care. These components include inter-professional communication, incentives for participation and performance and the adoption of new technologies and ways of working. These elements impact on “several groups and organisational levels” such as local and national commissioning bodies, primary and acute care in both the health and social care domains. As such, we locate the methodology within the broader theory of complex intervention evaluation and we draw from the UK Medical Research Council’s guidance [6].

This evaluation therefore has a deliberately broad focus to enable the different facets of the pilot’s consequences to be captured. A quantitative analysis, designed to measure activity—and consequently impact on cost—within the health system and analysis of changes to health outcomes. It also includes qualitative themes, looking at patient, clinician and manager experiences to the process of the implementation, the barriers to adoption, and wider questions of how the pilot fits into the national integration agenda. The four streams of the evaluation are described in Table 1, where methods of investigation match the core aims of the pilot. This evaluation will take place over the first year of the pilot, with further evaluation being planned for the future.

The ICP itself operates within a dynamic healthcare economy in the midst of financial challenges and national legislative changes [9, 10]. The underlying shape of the project has been subject to change and refinement including expanding to new locations and adjusting expectations in terms of its perceived outcomes and impact. This has made its evaluation a moving target [11]. We have therefore adapted our evaluation to these changes, to fit the on-going context.

In addition, the evaluation has not sought to remain separate from the pilot process, only publishing findings at a later date. Instead, information from the evaluation process is being fed back to the organisations taking part in the pilot, and to the pilot’s management board, regularly during the operation of the pilot. In particular, we will feed back findings to the ICP board in the form of an interim and a final report, and via several evaluation committee meetings en route, allowing the ICP management team to respond to finding so far (and potentially act to improve the intervention), and allowing the evaluation team to identify data sources and participants. The formative nature of this approach may lead to some confounding of our evaluation of the intervention; however the reality of service redesign is often that evaluators need to work closely with implementers.

## The evaluation framework

### Workstream 1: Impacts on service use and costs

One of the intended consequences of the NWL ICP is to change the pattern of service use, and in particular, reduce the use of more expensive hospital care by substituting better preventive and anticipatory care [12]. This element of the evaluation will look at the extent to which the pattern of health and social care service use has changed for patients.

In undertaking this work, we will seek to maximise the sample of cases under study by exploiting existing administrative information. Though this means that the results are influenced by the quality and depth of information recorded, the approach does have some advantages in that data collection is relatively inexpensive. Hence, it becomes possible to look at large sample sizes and that for individual-based analyses administrative data overcomes problems of recall bias when asking people about service use and medical diagnoses. Through linkage of administrative data sets, it is also possible to look at how patients use resources across organisations including social care [13].

The analysis looks at changes and difference in a number of metrics of activity. These are the number of hospital admissions, out-patient attendances and A&E attendances, the estimated costs of these events, intensity of social care service use (notional cost per person per month), number and estimated cost of GP visits and community nursing inputs (where data allow). Activity will be costed to weight different forms of activity using methods applied in previous work on national resource allocation models [14] and in studies of social care [15].

One of the key challenges in undertaking analyses of changes in hospital use for complex interventions is that individuals may be selected for an intervention because they have a high use of health services. The problem is that any subsequent fall in utilisation in this group may simply be due to regression to the mean—that is people reverting to a normal level of use irrespective of the intervention [16, 17]. This means that simple changes over time are not sufficient to show an effect but there needs to be some form of control group to show what would have happened anyway. Whilst randomised prospective analyses would overcome these problems, these were not feasible in this instance due to resource constraints. As an alternative, we plan to identify controls through a quasi-experimental design selecting from a wider population, a subgroup of matched controls that are sufficiently similar to the intervention group with respect to a set of baseline variables (age, sex, comorbidities and hospital activity up to point of intervention). The aim is to derive a control group that is well matched on all potential confounder variables so that a statistically valid inference can be drawn (see Box 2).

#### The proposed analysis will have two arms

##### a. Comparison of use of hospital services relative to an external control group

In this part of analysis, we will focus on a comparison with a control population drawn from other areas in England. Information will be available for the individuals enrolled in any intervention, and also for the whole populations of general practices which are participating in the Integrated Care Pilot. The data will be at person level but anonymised so that the research team cannot identify sensitive personal information or individual identities. The NHS Information Centre for Health and Social Care will act as a trusted third party to handle any confidential information and create the anonymised linked fields for use by the research team.

The aim will be to look at patterns of hospital use for this group compared to matched individuals taken from across the country representing changes associated with ‘usual care’. We will select five local authorities that are the most similar to the NW London population using Office for National Statistics (ONS) corresponding health areas methodology [22].

Information on the prior patterns of diagnoses and hospital utilisation will be used to stratify cases according to the risk of admission. The actual level of utilisation before and after the agreed starting point in the pilot will be compared. In this way, we will be able to track levels of hospital use for cohorts of people for 2–3 years before they became part of the pilot. We will then test for subsequent change and compare results by risk strata. Our analysis strategy is built around a generalized difference-of-differences regression approach at the person level.

This external comparison has the advantage that it enables more precise matching as it draws from a much larger pool of potential controls. It will also show how services within NWL cases have changed with respect to usual care in other areas. The main drawback is that this approach has to use information common to cases and controls—which effectively limits the analysis to routinely collected hospital based information on diagnoses and activity.

##### b. Comparison of other health and social care services change over time

In this arm, we are able to access much more detailed sets on health and social care usage for participants in the ICP. In the first instance, this allows us to document changes in utilization across sectors for a cohort of patients. This is useful in ascertaining the relative weight of different services in overall costs, and also indicating whether change in the use of one service is accompanied by change in another. So for example is a reduction in hospital bed days in the intervention group offset by an increase in social care use? There will also be scope to compare pre-post patterns of service use specifically linked with different start dates at practice level.

We will also explore the extent to which we can derive matched controls based in a wider range of local data sets—however this requires data for the whole populations to be accessible. For some data sets collections may be limited to specific cases only.

### Workstream 2: Impacts on clinical service quality (process and outcomes)

An important and innovative feature of the ICP is the availability of linked, patient-level primary, secondary, community health services and social care data to all the clinical teams involved, via an Information Portal which integrates these data sources. Data integration is being recognised as an important intervention in its own right in integrated care programmes. The same data is being used for the evaluation in anonymised form via a Data Sharing Agreement (informed patient consent is not required for the secondary use of data for clinical audit or NHS service evaluations). Six years’ retrospective primary care data and three years’ NHS Secondary Uses Service (SUS) data is available for analysis. This allows patients’ health and social care data to be tracked across sectors and over time, and also casemix adjustment for demographic and comorbidity covariates.

Because there is a long-standing trend towards improved management of chronic diseases in the NHS, simply showing that care improved after the introduction of an ICP would not be sufficient to demonstrate their effectiveness. We would need to know that the improvement was greater than that based on underlying trends and that the improvement was also better than in non-ICP settings. This requires data from before the introduction of the ICP and also comparing performance against a non-ICP site. Furthermore, as this evaluation is being carried out in London, the most socio-economically and ethnically diverse part of the UK, it is important that the ICP evaluation takes into account the characteristics of the populations the ICP serves. Also important is to see how well the ICP addresses the well-recognized socio-economic and ethnic disparities in access to health services and in health outcomes.

In addition to service utilization and costs, the evaluation will examine clinical effectiveness, both in terms of outcomes and process measures, for the two groups (elderly and with diabetes) covered by the ICP. The study will look at specific clinical process and outcome metrics, described in Table 2, using both time trends and a case comparison methodology at the practice level, comparing patient data before and after they received an ICP care plan and patients in ICP practices who are eligible for the ICP but have not yet been asked for consent or received a care plan. There will also be comparisons between local practices that have chosen to be a part of the pilot, with those who have not, at the practice level as patient-level primary care data is not available for the latter. Specifically, we will examine whether the introduction of the ICP has resulted in improvements in health outcomes for patients with diabetes and for older patients.

As the key aim of any health intervention is to improve quality of care, patient safety and clinical outcomes, these should be key measures in the evaluation. This means quantifying the process and outcomes of care. For many areas of health care, standards already exist (e.g. Quality and Outcomes Framework (QOF), National Institute for Health and Clinical Excellence (NICE) guidelines). Examples would include HBA1c, blood pressure and cholesterol control in people with diabetes. Other key areas for quantitative evaluation are patient experience and impact on NHS efficiency and costs. Impact on NHS efficiency would include areas such as unplanned admissions for ambulatory-sensitive conditions, A&E attendances, and inappropriate prescribing, all focusing on specific diseases to improve sensitivity, although this must be traded off against reductions in numbers of events. We are also aiming to measure changes in care processes covered by the ICP Care Packages, for example, referrals to fall services.

Apart from regarding mortality and utilization of unscheduled care as adverse endpoints, there is a dearth of available outcome data meaningful to patients. The use of disease-specific patient-reported outcome measures (PROMs) in primary care is the subject of an Oxford pilot funded by the Department of Health. However the use of a measure of health-related quality of life, such as the EQ-5D, by ICPs would be of great utility both for clinical care and evaluation.

In the statistical analysis, we will compare percentage differences in annual measurement of the outcome measures using χ^2^-tests. Linear regressions for pre-ICP data for each patient will be generated with a time indicator, and the slope and intercept will be used to predict the future value. This value represents the expected value of the outcome if the ICP had not been established. An additional challenge in the statistical analyses will be to accommodate the hierarchical nature of the data, which are years of measurement nested within patients nested within practices. Ignoring this multilevel clustering would result in faulty estimation of standard errors. We will therefore use a random effects multilevel model in this analysis to adjust for casemix at the practice level.

A spatial analysis will be also conducted using a Geographic Information Systems (GIS). Patient data will be mapped at the Lower Super Output Area (LSOA) to explore the spatial distribution of patients enrolled in the ICP compared to controls. Similarly maps will be created which display the geographic distribution of outcomes, both at practice level and aggregated from individual or practice level data as median values to LSOA level across the ICP area pre- and post-intervention. The mapping will assist in identifying geographic areas where there is higher uptake of the ICP and allow for monitoring of outcomes over time and space to detect where outcomes are affected by spatial factors.

### Workstream 3: Qualitative assessment of the impact of the Pilot

This part of the study investigates the human perception, experience and involvement of participants in the pilot. We hope to develop a comprehensive understanding of the patients’, carers’ and professionals’ experiences and perceptions of the pilot, as well as their suggestions for effective implementation. The main areas of interest are 1. Patient perception of continuity of care; 2. Patient perception of changes in clinical decision making; 3. Provider experience of communication between professional groups; and 4. The role of Multidisciplinary group meetings in the integrated care change management process.

In addressing these broad objectives, we are employing a mixed methods design. This will include focus groups with patients and professionals to understand their perceptions and experiences of the pilot and semi-structured interviews with a purposive sample from both patients and professionals to investigate the perceptions of all users. Topic guides for these interviews focus on users’ experience of the integrated pilot, and their attitudes towards the intervention. We will use a thematic approach to explore and integrate the findings of this approach [23].

To complement these findings, we will also develop and implement a survey to record patient and carer experience, and a separate survey of professional experiences. Survey questions will explore issues such as motivation to take part in the pilot and experience of participating, but will also incorporate questions specific to themes raised from participant interviews and observations, to ensure the surveys reflect the challenges faced by the pilot during implementation.

Finally, in this qualitative component, we will include a novel analysis of patterns of communication within MDG meetings, looking at the nature and direction of conversation between participants. MDG meetings involve the participation of GPs, hospital consultants, community and social service professionals each from different organizations within the local health economy and are therefore different to MDGs within hospital settings. We will explore whether traditional power relationships and communication patterns exist and persist or are broken down leading to a more integrated way of working between the professional groups. Does the discussion of the complex clinical cases brought to the MDG meeting lead to or foster opportunities to consider the wider health economy and ways to improve and identify efficiencies in and between participants’ respective organizations? This will involve recording and transcribing multidisciplinary group meetings, and then coding the utterances that occur. We draw on Bale’s validated coding scheme [24] to describe and characterize the content of the utterances and the kind of interactions that are occurring. We will be exploring whether some professional groups dominate the conversations and whether their discussions focus on individual patient level detail or broader ways of working together as a heterogeneous but integrated group. A more full description of the methodology of this novel approach is available elsewhere [25].

The findings from this component of the evaluation will add to our understanding of professional practice in integrated care programmes and contribute towards a framework of knowledge to inform policy and organisational change processes related to integration, enhanced communication and collaborative working.

### Workstream 4: Strategic evaluation of the pilot within the national policy context

The final part of the evaluation has the broadest focus; examining how the pilot operates at an organisational level and how the wider policy environment has shaped the design and implementation of the initiative. This component also aims to ground the evaluation of the pilot in the context of the field of integrated care in the NHS and beyond. This workstream complements the other workstreams by taking a strategic overview. To this end, we aim to explore how national policy has impacted upon the design, implementation and operation of the pilot, identifying factors that have facilitated or hindered progress. By locating the pilot within the wider literature and evidence base, we hope to draw insight from other national and international models of integrated care, identifying and exploring areas where the pilot appears to be distinctive. We will also examine how the pilot is developing at a strategic level in order to understand the organisational level motivations for, and challenges of, developing integrated care.

This part of the evaluation will be addressed via a programme of on-going policy analysis, observations and semi-structured interviews. Interviews are being undertaken with senior representatives of the key organisations involved in the pilot. We seek to understand the organisational and strategic motivations for engaging with the pilot and any challenges and barriers to doing so. Interviews also aim to understand how national policy and local contextual factors—such as organisational relationships, financial positions and local priorities—have shaped the design of the pilot and helped or hindered its implementation and development. On-going consultation with key individuals in the wider policy arena will ensure that interviews with those involved in the pilot address appropriate issues. The policy literature will be regularly reviewed to ensure that new evidence and emerging issues are taken into account.

Interviewees from all key organisations have been identified to ensure the evaluation understands the pilot from the perspective of all the different players. A number of observations of board meetings, committee meetings and operational team meetings will complement interviews by offering an insight into how the pilot is being implemented and highlighting particular dynamics and challenges at a strategic level. Interview and observational data will be analysed by qualitative researchers who will identify key themes, drawing out the most important barriers and enablers. The framework of analysis will be based on the theoretical literature of integrated care and the wide body of policy literature. Where appropriate, comparisons will be made with other examples of integrated care. This analysis will add to the body of literature and evidence on the implementation of integrated care initiatives, extending our understanding of the challenges involved in executing large scale change within a dynamic policy environment.

## Discussion

### Dealing with complexity

The complex nature of the intervention and the environment will make attribution of cause and effect difficult. This pilot is occurring in a period of almost unparalleled structural reforms in the English National Health Service—many of which may have an impact on the desired effect of the pilot. The ICP could be described as the introduction of a complex intervention into a complex environment—having the characteristics of adaptation and learning by both those delivering and receiving the intervention, feedback loops, a sensitivity to starting conditions and with a diversity of activities and emergent outcomes [11, 26].

We are aware of the tension between providing early evaluation results to inform decision makers—against the need to undertake rigorous analytical methods. For example, in some cases changes can only be confirmed with large samples and longer follow-up periods. In addition, experience from other countries shows that successful integrated care organisations take many years or decades to develop.

Exploring the counterfactual in this case will consequently be difficult. The presence of control groups is reassuring, but comparison against other areas of a national health economy where innovation is being actively encouraged means that it is difficult to confirm if the control groups are genuinely intervention free. At best, they represent the average pattern of care seen outside of this pilot environment. There are also other projects in the same geographical area that might influence the findings. Year on year comparison is made more difficult by the secular trends and on-going structural changes occurring in the NHS. Furthermore, any evaluation that includes clinical outcomes has to be timed appropriately, to allow the natural history of disease and the effect of interventions on this to take its course. Although this plan describes a time limited evaluation, relating to cost, an ideal evaluation would follow patients and appropriate controls over a prolonged period. Given this environment and the complex and iterative nature of the intervention, the evaluation requires a degree of flexibility in method and process—to learn and adapt as the intervention does so.

We accept that there are limitations to our approach, and that we were not able to move systematically though the various stages of evaluating a complex intervention that would ideally be done using the MRC’s framework, including modelling and delivering a small scale proof of concept. However, given the financial constraints placed on many innovative delivery models in the current period of financial austerity, we suggest that this work will provide a useful, real world model for others attempting the evaluation of similar schemes.

Strategies we have adopted in the evaluation design to mitigate against the various challenges faced are listed in Table 3. We appreciate that there are many attempts to evaluate complex interventions using mixed methods approaches. We believe that this approach adds extra depth, beyond simple quantitative aspects of performance and qualitative assessment of user experience, by measuring integration behaviour and strategic, organisational level experience. This may serve as a useful resource for others also embarking on the evaluation of complex interventions such as integrated care pilots.

## Conclusion

The proposed methodology, with a focus on looking at service usage and quality, and a matched case comparison approach will allow a robust assessment of effectiveness of a large integrated care intervention within the NHS. We believe that the investigation of the qualitative aspects, including ways of working, barriers to adoption and staff and patient experience, will allow us to gain an insight into those ‘softer’ cultural aspects of the development of the integrated care model, which have often proved so hard to do.

Despite its limitations, we hope this evaluation allows us, in the words of Tom Ling, to clarify contribution (how reasonable is it to believe that the intervention contributes to the intended goals effectively) even if it is not able to definitely identify attribution (what proportion of the desired outcomes was produced by the intervention) [11]. The broad, dynamic and responsive nature of the approach should allow some of the inherent complexities to be accounted for. Given the priority given to the integrated agenda by governments internationally, the need to develop and improve strong evaluation methodologies remains strikingly important.

## Acknowledgements

The Department of Primary Care & Public Health at Imperial College London is grateful for support from the NW London NIHR Collaboration for Leadership in Applied Health Research & Care (CLAHRC), the Imperial NIHR Biomedical Research Centre, and the Imperial Centre for Patient Safety and Service Quality (CPSSQ). The views expressed in this publication are those of the authors and not necessarily those of the NIHR, BRC or CPSSQ.

## Funding

The evaluation of the NWL ICP is funded by the Imperial College Healthcare Charity.

## Conflicts of interest

The authors declare no conflicts of interest.

## Reviewers

**Steve Iliffe**, Professor of Primary Care for Older People, UCL, Dept of Primary Care & Population Health, Rowland Hill St., London NW3 2PF, UK

**Sarah Purdy**, Dr. Reader in Primary Health Care, Centre for Academic Primary Care, NIHR School for Primary Care Research, School of Social and Community Medicine, University of Bristol, Canynge Hall, 39 Whatley Road, Bristol, BS8 2PS, UK

Two anonymous reviewers.

## Figures and Tables

**Table 1.**
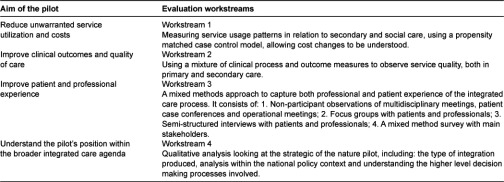
What this Integrated Care Pilot evaluation looks at.

**Table 2.**
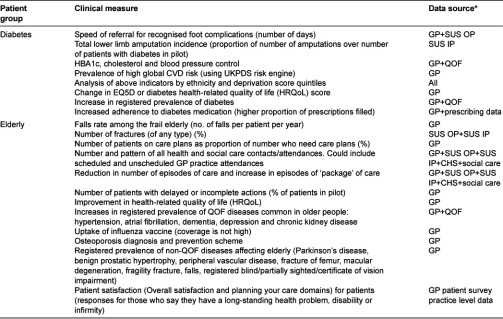
Clinical outcome and process measures.

*GP: General Practice record, SUS IP: Inpatient Secondary Uses Service Data, SUS OP: Outpatient Secondary Uses Service Data, QOF: Quality and Outcome Framework.

**Table 3.**
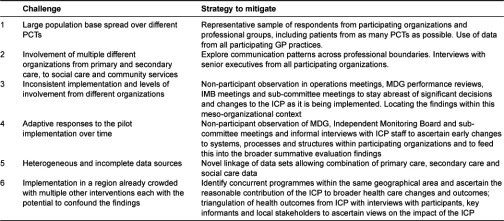
Challenges and responses of this evaluation approach.

**Box 1:**
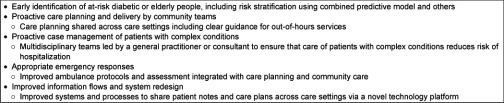
Interventions in the North West London Integrated Care Pilot (adapted from Harris et al. 2012) [8]

**Box 2:**
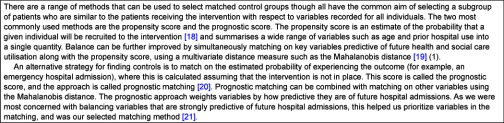
Approaches to identifying matched controls.
